# A Simple and Low-Cost Optical Fiber Intensity-Based Configuration for Perfluorinated Compounds in Water Solution

**DOI:** 10.3390/s18093009

**Published:** 2018-09-08

**Authors:** Nunzio Cennamo, Girolamo D’Agostino, Filipa Sequeira, Francesco Mattiello, Gianni Porto, Adriano Biasiolo, Rogério Nogueira, Lúcia Bilro, Luigi Zeni

**Affiliations:** 1 Department of Engineering, University of Campania Luigi Vanvitelli, 81031 Aversa, Italy; f.mattiello@gmail.com (F.M.); luigi.zeni@unicampania.it (L.Z.); 2 Copernico S.r.l., 33100 Udine, Italy; girolamodag@gmail.com (G.D.); porto@copernicon.it (G.P.); biasiolo@copernicon.it (A.B.); 3 Instituto de Telecomunicações, 3810-193 Aveiro, Portugal; fsequeira@av.it.pt (F.S.); rnogueira@av.it.pt (R.N.); lucia.bilro@av.it.pt (L.B.)

**Keywords:** plastic optical fibers (POFs), molecularly imprinted polymers (MIPs), PFOA (Perfluorooctanoate), PFAs (Perfluorinated Alkylated Substances), chemical sensors, optical fiber sensors

## Abstract

We present a very simple approach for the detection of the Perfluorinated Alkylated Substances (PFAs) in water solution. Perfluorooctanesulfonate (PFOS) and Perfluorooctanoate (PFOA) are the most extensively investigated perfluoroalkyl and polyfluoroalkyl substances in water because human exposition can occur through different pathways, even if the dietary intake seems to be their main route of exposure. The developed sensor is based on a specific Molecularly Imprinted Polymer (MIP) receptor deposited on a simple D-shaped Plastic Optical Fiber (POF) platform. This novel chemical sensor has been characterized using a very simple and low-cost experimental setup based on an LED and two photodetectors. This optical sensor system is an alternative method to monitor the presence of contaminants with an MIP receptor, instead of a surface plasmon resonance (SPR) sensor in D-shaped POFs. For the sake of comparison, the results obtained exploiting the same MIP for PFAs on a classic SPR-POF sensor have been reported. The experimental results have shown that the actual limit of detection of this new configuration was about 0.5 ppb. It is similar to the one obtained by the configuration based on an SPR-POF with the same MIP receptor.

## 1. Introduction

Optical fiber bio/chemical sensors have been shown to be able to play an important role in many fields [1,2,3,4,5,6]. In recent years, plastic optical fibers (POFs) have been well known for their successful use as optical fiber sensors. POFs show higher resistance to harsh environments, flexibility, simpler manufacturing and handling procedures, great numerical aperture, and allow easy connectorization due to the large diameter of the fibers. These fibers share some attributes with glass optical fibers, such as immunity to electromagnetic fields, small size and weight, but also allow the production of low-cost sensing systems. Bilro et al. presented a review of POF sensors technology with a special focus on intensity variation schemes and low-cost solutions [7]. Jin and Granville reviewed the recent progress in POF sensors, focusing on intrinsic detection schemes [8]. In fact, the optical fiber sensors can be classified as intrinsic and extrinsic, depending on whether the fiber is interacting with the analyzed medium or if it is used only as a waveguide that allows the propagation of the light to the sensing region and its collection, respectively. The detection scheme can be based on reflection, where the light source and detector are placed on the same side of the fiber; or, on transmission, if they are placed on opposite sides. In both detection schemes, there are several transduction mechanisms that can be employed, including sensors based on variations of the evanescent field or on spectroscopic methods (absorption, fluorescence and refractive index).

Cennamo et al. presented a simple and low-cost approach to plasmonic sensing exploiting a sensing multilayer deposited on a D-shaped POF, combined with a very easy experimental setup based on a white light source and a spectrometer [9]. In the last five years, Cennamo et al. implemented important applications by specializing the same plasmonic POF platform with different kinds of receptors, such as Molecularly Imprinted Polymers (MIPs) and Bio-receptors [10,11,12,13].

Recently, Sequeira et al. presented a D-Shaped POF platform for refractive index sensing through an intensity-based configuration [14], which revealed the suitability of chemical sensing through an evanescent field with a simple and low-cost POF sensor. In this work, a synthetic receptor (MIP), specifically designed to recognize Perfluorinated Alkylated Substances (PFAs) in water, is used for the first time with this intensity-based D-shaped POF platform.

PFAs have been widely used for the last fifty years in many industry sectors and their dispersion in water has been recognized as highly dangerous for eco-systems, biodiversity and human health. The EU directive 2013/39/UE is listing PFAs among the priority substances to be completely eliminated within the next 20 years, thus making this issue extremely urgent.

These contaminants are very persistent and refractory to different biological and chemical treatments and their presence in an environmental matrix can give rise to toxic and bio accumulative effects, particularly to mammalian species. Immune-toxic effects of PFAs to cellular systems and animals are largely demonstrated, and different epidemiologic research studies have shown the potential effects of these chemical compounds on various human immune diseases [15,16,17].

The conventional proposed analytical methods are based on chromatographic techniques coupled with mass spectrometry. Furthermore, sensors based on electrochemical and colorimetric approach have also been described. All of the mentioned methods are time-consuming, expensive and they often require a non-easy pre-treatment step [18,19,20,21,22]. In order to beat these drawbacks, it is needed to find a rapid, simple and sensitive method for the detection of perfluorinated alkylated substances. 

In perfluorooctanoic acid (PFOA) and Perfluorooctanesulfonic acid (PFOS) detection, a very attractive perspective is the use of a platform based on optical fibers for fast, in situ and/or remote-controlled detection. Bio and chemical sensors using optical fibers have been shown to be able to play an important role in refractive index (RI) sensing, especially where fast, portable, low cost and durable units are needed for early detection and identification [1,2,3,4,5,6,7,8,9]. On this line of argument, we exploited a simple and low cost chemical sensor based on a D-shaped plastic optical fiber platform together with a novel biomimetic polymer for the detection of PFAs in aqueous medium. A molecular imprinting technique is a convenient tool for the preparation of molecular-recognition materials characterized by good chemical stability and selectivity. Molecularly imprinted polymers (MIPs) are biomimetic materials imprinted with a template molecule for the purpose of retaining a memory of that specific analyte (or a specific class of molecules). MIPs are synthetic receptors with many favourable aspects with respect to bio-receptors, such as an easier and faster preparation, the possibility of application outside the laboratory, for example under environmental conditions, a longer durability [23,24,25]. Moreover, the advantage of MIPs is that they can be directly deposited on a flat sensing surface by a spin coater machine.

According to the above considerations, an MIP, specifically designed to recognize PFAs in water, is deposited on the core of a D-shaped POF platform, by a spin coater machine, to perform intensity based measurements. This synthetic receptor is designed to recognize C4 to C12 PFAs, even if we will show only PFOA response. Actually, we already demonstrated, exploiting the SPR approach, that the MIP response is similar to PFOA or C4 to C12 PFAs [26]. Thus, for the sake of comparison, we deposited the same MIP receptor on an SPR-POF configuration, as already done in [26]. In summary, in the present work, we show two transmission POF chemical sensors, both based on the same MIP receptor for PFAs, one based on evanescent field sensing and another one (reference sensor [26]) on plasmonic sensing. 

The obtained results have shown good performance in terms of sensitivity and limit of detection (LOD) of this novel approach. The LOD of this sensor system, obtained by approximating the Hill equation to the Langmuir equation [26,27,28], is completely comparable to the configuration based on a surface plasmon resonance (SPR) sensor in D-shaped POF (0.13 ppb) [26], but with the advantage of an easier and low-cost preparation procedure and experimental setup.

## 2. Materials and Methods

### 2.1. Receptor Layer

The MIP receptor exploited in the present work is the same used in [26]. Nevertheless, in order to make this paper self-contained, we reported, in the following, all the information about the MIP preparation along with those relative to the optical sensor’s configuration.

#### 2.1.1. Chemical Reagents

(Vinylbenzyl) trimethylammonium chloride [26616-35-3] (VBT), 2,2-azobisisobutyronitrile [78-67-1] (AIBN), 1H,1H,2H,2H-Perfluorodecyl acrylate [27905-45-9] (PFDA), ammonium perfluorooctanoate (FPO-NH4) [3825-26-1] were obtained from Sigma–Aldrich (Saint Louis, MO, USA) and used without any further purification. Ethylene glycol dimethacrylate [97-90-5] (EDMA) (Sigma–Aldrich cod. 335681, Sigma–Aldrich, Saint Louis, MO, USA) was distilled in vacuum prior to use in order to remove stabilizers.

All other chemicals were of analytical reagent grade. The solvent was deionized water. Stock solutions were prepared by weighing the solids and dissolving in ultrapure water (Milli-Q).

#### 2.1.2. Pre-Polymeric Mixture: Preparation and Deposition

The pre-polymeric mixture of the MIP was prepared with ammonium perfluorooctanoate (FPO-NH4) as the template, VBT and PFDA as the functional monomers, EDMA as the cross-linker and AIBN as the radicalic initiator. The reagents were mixed at the following molar ratio 1 (Template):4 (VBT):5 (PFDA):50 (EDMA). The mixture was uniformly dispersed by sonication (visually homogeneous milky solution). Deionized water was added to dissolve all reagents (volume ratio H2O:EDMA = 1:17.5). Finally, the AIBN was added to the solution in non-stoichiometric ratio. 

The MIP layer was deposited as hereafter described. The D-shaped sensor was placed in the spin coater. About 100 μL of the pre-polymeric mixture were dropped over the D-shaped sensing region and spun for 1 min 20 s at 800 rpm, after which the sensor was placed in the oven and the thermal polymerization was carried out at 72 °C for about 16 h. 

The obtained polymeric film was washed and the template molecule was extracted, leaving the imprinting sites free for rebinding.

The washing and extraction procedures were performed in two steps. In the first step (washing), the MIP layer was washed with 96% *v*/*v* ethanol in order to remove not-polymerized monomers’ residue. In a second step (extraction), the template was extracted from the MIP by washing with HCl solution (2% *w/w*) and 96% *v/v* ethanol. The first step is conducted pouring 5 mL of ethanol on the platform and second step pouring 1.5 mL of HCl solution, 5 mL of ethanol, 1.5 mL of HCl and 5 mL of ethanol. Finally, the sensor was poured with deionized water and dried at room temperature.

### 2.2. D-Shaped POF Sensor: Intensity-Based Configuration

Polymeric optical fibers (POFs) with 1 mm diameter from Asahi Kasei (DB-1000, Plastic Optical Fiber Marketing & Development Group, Tokyo, Japan), were selected with the following characteristics: a polymethyl methacrylate (PMMA) core of 980 µm, a fluorinated polymer cladding, a numerical aperture of 0.5 and a refractive index in the visible range of interest of about 1.49 for PMMA and 1.40 for the fluorinated polymer.

To obtain the optical platforms, as extensively reported in [14], the POFs were prepared in 20 cm segments with a fiber optic cutter from Rennsteig and embedded in 6 cm length grooves engraved on planar holders (size: 6 cm long, 1 cm high and 1 cm wide). These planar platforms used in this study were made with ABS plus™ production-grade thermoplastic, produced with Mojo® 3D Printer, provided by Stratasys^®^ FDM^®^ (Eden Prairie, MN, USA). The groove dimensions were 1 mm in width and 700 µm in depth, with an instrumental error of 100 µm. For the polishing and spinning steps, we handle the sensors through these holders [14].

**Figure 1:**
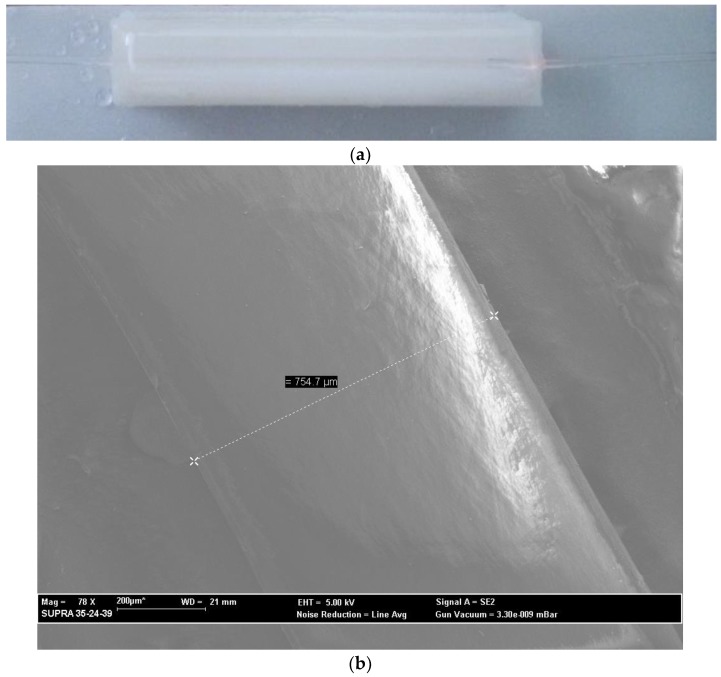

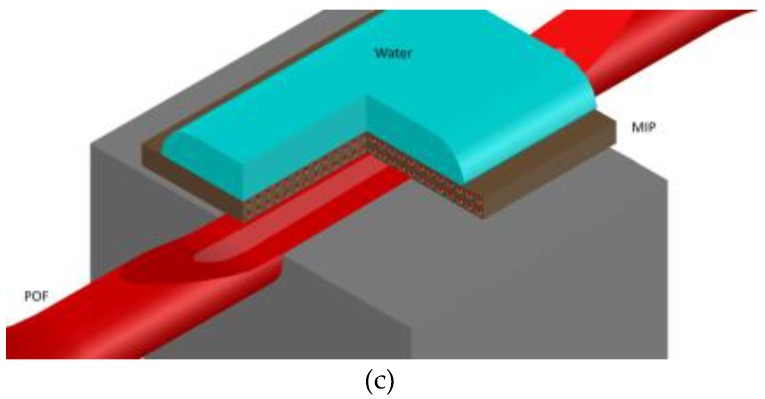
POF sensor: (**a**) picture of the D-shaped POF sensor platform; (**b**) typical SEM image of the optical platform; (**c**) sensing region outline of a D-shaped POF with an MIP receptor.

Figure 1a shows a picture of the sensor platform. The surface of the POF embedded in the block was polished with sandpaper of 22 µm (P800) with an “8-shaped” pattern about 30 times in order to remove the cladding and part of the core. We count the number of rounds on the polishing papers and, finally, we check the D-shaped area by SEM (scanning electron microscopy), as reported in [14]. A typical SEM image is reported in Figure 1b.

The polishing process is very important in the performances of the sensor platform. This analysis has been widely reported in several works [26,29].

The desired D-shape was obtained. As shown in Figure 1c, the planar surface (D-shaped area) can be employed for depositing the MIP layer, as we will explain in the following section, by a spin coater machine.

The sensing region of the optical platform with the MIP layer is represented in Figure 1c, with an interaction length of about 6 cm, whereas the estimated thickness of the MIP layer is less than one micron.

In this work, the same MIP has been deposited on several D-shaped platforms to test the repeatability. Furthermore, to compare the sensor performances with those of a different optical sensing method, we have deposited the same MIP on an SPR D-shaped POF (similar to [26]). We will report, in the next section, just one PFOA measurement for the intensity-based D-shaped platform and one for the SPR-based D-shaped platform.

**Figure 2:**
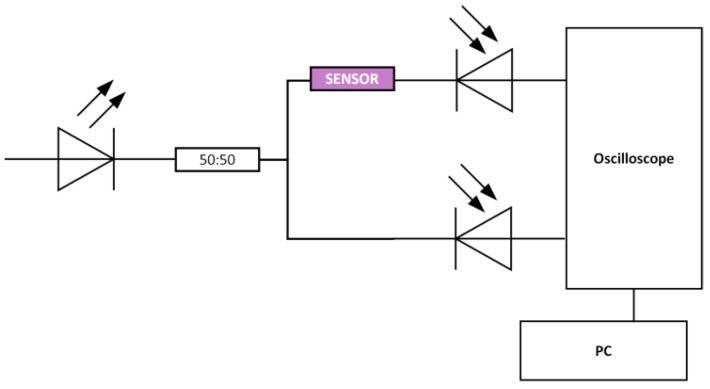
Outline of sensing setup.

The intensity-based D-shaped-POF-MIP sensor has been characterized using a simple and low-cost experimental setup based on an LED, 627 nm (Avago SFH757V, Broadcom Limited, San Jose, CA, USA), an optical coupler (50:50), two photodetectors (Avago SFH250V, Broadcom Limited, San Jose, CA, USA), and a digital low-cost oscilloscope (Picoscope, Pico Technology, Cambridgeshire, UK) connected to a laptop, as shown in Figure 2. 

Output data, time and voltage of the reference and sensor signals, in mV ($V_{reference}$ and $V_{sensor}$, respectively), were logged into a PC by means of Picoscope’s software. The self-referenced transmitted signal, k, was used to correct source fluctuations and variations due to external conditions, as defined in Equation (1):

(1) $$k=\frac{V_{sensor}}{V_{reference}}$$

### 2.3. Reference Sensor: Plasmonic Platform in a D-Shaped POF

**Figure 3:**
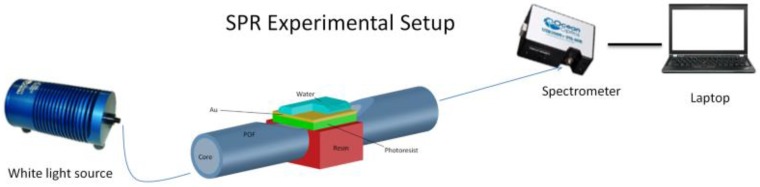
SPR sensor platform based on D-shaped POF [26].

An SPR fiber sensor has also been used to compare the performance of the new approach with a “classic” plasmonic approach [9]. Figure 3 shows the SPR-POF sensor with the experimental setup. The plasmonic platform was realized as described in [9,26]. In this SPR sensor, the length of the sensing region is about 10 mm. The photoresist layer is 1500 nm thick and the gold film is 60 nm, presenting a good adhesion to the substrate too. In this configuration (SPR-POF sensor), we have used a more expensive “spectral interrogation” [9,26]. Here, the experimental setup consists of a halogen lamp (HL-2000-LL, Ocean Optics, Dunedin, FL, USA) illuminating the sensor and a spectrum analyzer (USB2000+UV–VIS spectrometer, Ocean Optics, Dunedin, FL, USA) connected to a computer, as shown in Figure 3. The spectral emission of the lamp ranges from 360 nm to 1700 nm and the spectrometer is sensitive from 300 nm to 1050 nm.

## 3. Results

The sensor configuration was characterized with the same procedure used with the SPR platform [26], as it will be discussed below. In particular, after each addition of the sample (solution with different concentration of the analyte), a standard measuring protocol was implemented based on the following three steps: first, incubation step for chemical-interaction between analytes and MIP receptor (for 10 min at room temperature); second, washing step with water (blank); third, recording step for the data, when water (blank) is present as the bulk. This protocol is necessary in order to measure the response determined by the specific binding (analyte/receptor interaction) on the sensing surface, and not by the changes of the bulk refractive index or by non-specific binding between the surface of the sensor and the analyte.

All the measurements were performed in a laboratory at room temperature (23 °C). Possible temperature fluctuations during laboratorial characterization were filtered due to the self-reference methodology applied to the signal output. This shows the versatility and ability of the sensor to be applied in real field conditions.

This synthetic receptor was designed to recognize C4 to C12 PFAs at low concentration, even if we have shown only the PFOA response. We already demonstrated, by several approaches, that the MIP response is similar to PFOA or C4 to C12 PFAs, as reported in [26]. Using only PFO^−^ as the template, the recognition is focused on C8 but at a low concentration (lower than 2 ppb), the polymer can absorb PFAs between C4 to C12. The recognition is due to the ionic interaction common to all PFAs (carboxylic and sulphonic anion) and van der Waals interactions due to the -F groups. The amount of perfluorinated carbons is determined by the length of the C-C chain. The change in the refractive index, determined by the number of -CF2- groups, is such as to allow a similar response within the range of interest to be obtained. Column tests have allowed us to verify the possibility of moving the adsorption range in different windows of interest.

The produced D-shaped MIP-POF sensor was characterized with PFOA solutions with increasing concentration, from 0 ppb (blank) to 200 ppb. Each test started by adding deionized water to the sensor and signal output ($k_{norm}$) was obtained according to Equation (2):

(2) $$k_{norm}=\frac{k_{solution}}{k_{Blank}}$$

About 1.6 mL of solution without analyte (blank) was dropped over the sensing region, which was kept in a perfectly horizontal position and the data recorded for 1 min (after 10 min of incubation time and after the washing step). The mean value of the referenced signal (k) with respective Mean Absolute Relative Error (MARE), δk, was calculated with MATLAB software (R2015a, MathWorks, Natick, MA, USA).

**Figure 4:**
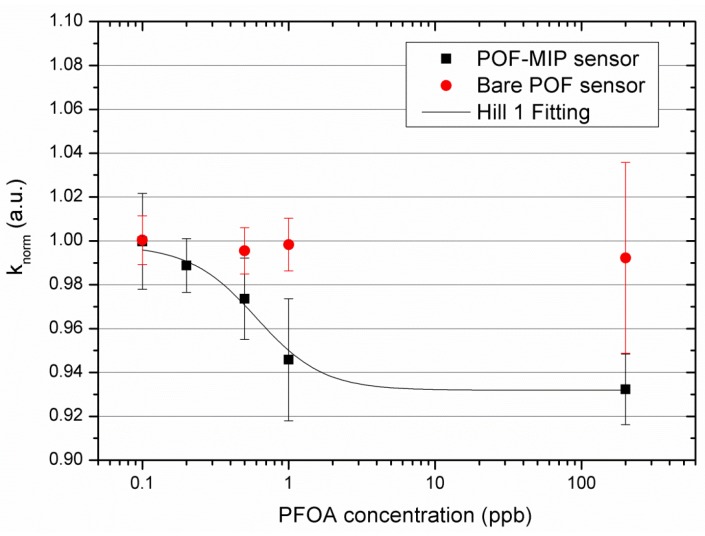
Response variation (respect to 0 ppb PFOA) versus the PFOA concentration (c), in semi-logarithmic scale, with the Hill Fitting, for the characterized D-shaped POF sensors (with and without the MIP layer).

Figure 4 shows the response of the D-shaped POF sensors with respect to 0 ppb of PFOA (blank), with and without the MIP layer, versus the PFOA concentration (ppb), in semi-logarithmic scale, given by the variation of the normalized signal ($k_{norm}$) and respective error ($\delta k_{norm}$). The Hill Fitting of the experimental values is also shown. OriginPro (2015, OriginLab Corporation, Northampton, MA, USA) was used to calculate the normalized signal and the respective error, and perform the Hill fitting. Table 1 lists the obtained values for both the characterized sensors (with and without the MIP layer). 

In this work, we present the results obtained with the PFOA compound in water (even if the response is similar to PFOA or C4 to C12 PFAs, as reported in [26]). In particular, when the concentration of the analyte increases, the output transmitted signal decreases. In this case, when the analyte concentration increases, the MIP refractive index (acting as the cladding in the D-shaped region) decreases, and the guiding characteristics of the optical waveguide change. 

On the other hand, when the sensor configuration without an MIP layer was characterized with the same concentrations of PFOA, no significant variation in the transmitted signal was observed. We suppose that this negligible variation has been caused by a minimal adsorption of PFOA in the PMMA POF core. The above result indicates that, for the D-shaped POF-MIP sensor, the binding between the MIP receptor and the analyte (PFOA) is present and that the signal variation is not due to an unspecific binding between the analyte and the surface of the POF’s core. 

It is important to underline that several similar sensor configurations have been considered, in order to perform a repeatability test, and all of them gave the same results.

Figure 4 shows that the experimental data obtained for the D-shaped POF-MIP sensor are well fitted by the Hill equation (Equation (3)), reported below:

(3) $$k_{norm}=k_{start}+\left(k_{end}-k_{start}\right)\frac{c^{n}}{K_{Hill}^{n}+c^{n}},$$

where $k_{start}$ is the value obtained without PFOA (blank) and $k_{end}$ is the plateau value obtained at high concentrations. The symbol c indicates the concentration of the analyte and $k_{norm}$ indicates the output at c PFOA concentrations. The two parameters $K_{Hill}$ and n are here considered simply as descriptors of the standardization curve, but they can also have a physical meaning. 

The Hill fitting has been obtained through OriginPro software and the parameters obtained with the associated standard errors are listed in Table 2.

From Equation (3), it is possible to notice that, if n ≈ 1 and at low concentration, i.e., at c much lower than $K_{Hill}$, the dose-response curve is linear, with sensitivity $\left(k_{end}-k_{start}\right)/K_{Hill}$, defined as the “sensitivity at low concentration”, as shown in Equation (4):

(4) $$k_{norm}-k_{start}=\frac{\left(k_{end}-k_{start}\right)}{K_{Hill}}\cdot c=\frac{\Delta k_{max}}{K_{Hill}}\cdot c$$

## 4. Discussion

A standard curve like that reported in Figure 4, with the Hill parameters listed in Table 2, is commonly used for chemo and biosensors, and their physical meaning can be related to the absorption due to the combination of the template at specific sites, when the number of receptor sites available for the combination with the substrate is limited. In that case, the adsorption takes place according to the Langmuir absorption isotherm [27]. The value of n in the Langmuir sorption model should be 1, and here it can’t be considered as significantly different from 1. 

The parameter $K_{Hill}$ of the Hill equation (Equation (3)) corresponds to the reciprocal of the affinity constant of the specific sites of the Langmuir model, whereas the limit of detection (LOD) can be calculated as the ratio of three times the error of the blank ($\delta k_{start}$) and the sensitivity at low concentration ($\Delta k_{max}/K_{Hill}$). Table 3 shows the sensitivity at low concentration and the LOD of PFOA detection in water by a D-shaped POF-MIP intensity-based configuration. 

The LOD value of 0.21 ppb has been obtained by approximating the Hill to Langmuir equation and used to compare the obtained LOD value with other LODs relative to different configurations. However, as the actual experimental results are concerned, Table 1 and Figure 4 reveal that the error bars on the data points relative to the D-shaped POF (without MIP) and the D-shaped MIP-POF overlap to some extent. Thus, in sensor applications, it is reasonable to consider 0.5 ppb as the actual LOD of this configuration.

To compare the performances of this novel low-cost sensor system with a “reference sensor”, we reported the results obtained by a “classic” configuration based on an MIP receptor deposited on an SPR-POF platform [9]. Similarly, to the experimental results presented in [26], we have incubated solutions at increasing concentrations of PFOA in water, in the range of interest (0–4 ppb). 

In order to verify the binding between the MIP sensing layer and analyte, the response of SPR-POF platform with an NIP (non-imprinted polymer) layer was also reported. In this case, the composition was the same as previously described but without adding any template, in order to obtain an NIP layer, as extensively discussed in [26].

**Figure 5:**
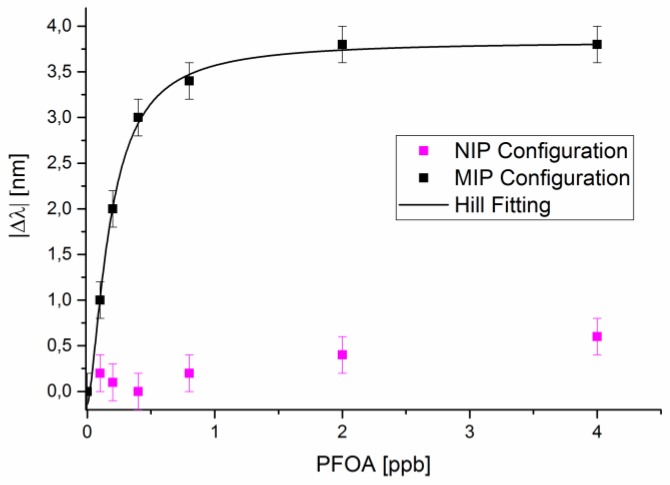
For MIP and NIP SPR-configurations, absolute plasmon resonance wavelength variation (|∆λ|), with respect to the blank (0 ppb), versus the concentration of PFOA (ppb) and Hill fitting to the experimental values (in MIP configuration).

For both NIP and MIP configurations, Figure 5 reports the absolute resonance wavelength shift, (|∆λ|), with respect to the blank (PFOA 0 ppb), versus PFOA concentration (0–4 ppb), along with the Hill fitting to the experimental data for the SPR-POF-MIP configuration. Each experimental value is the average of five subsequent measurements and the respective standard deviations (error bars) are shown. When the PFOA concentration increases, for the SPR-POF-NIP configuration, the shift of the resonance wavelength is not present. For the SPR-POF-MIP configuration, the resonance wavelength shifts to smaller values when the concentration of PFOA in water solution increases. A shift like this means that when the PFOA interacts with the MIP receptor, the refractive index value of the MIP layer decreases [26]. This phenomenon is also present when the PFOA interacts with the antibody (bio-receptor) on the same SPR-POF platform [28].

From the Hill fitting of data reported in Figure 5, as illustrated in [26], we have calculated the chemical parameters, reported in Table 4, obtained with the same MIP for PFOA deposited on a D-shaped POF-SPR sensor. Table 4 clearly shows that the LOD obtained with a D-shaped POF-MIP sensor using an intensity-based configuration is comparable to the value obtained using a surface plasmon resonance technology (SPR-POF sensor).

## 5. Conclusions

In this work, a simple evanescent-wave platform, based on a D-shaped POF sensor, has been used for the first time with an MIP receptor. In particular, we have developed and characterized an optical-chemical sensor for PFAs detection using a low-cost intensity-based setup. 

The performances of the proposed system are comparable to those obtained by a “classic” SPR-POF platform combined with the same MIP receptor, but with an easier and low-cost preparation procedure and an experimental setup only based on an LED and two photodetectors. 

This shows a promising future for the development and optimization of low-cost POF chemical sensors for water quality assessment. In fact, we can consider the actual LOD of the sensor as 0.5 ppb, a value that is compatible with the maximum residue limit fixed by the European Union regulations in the monitoring of PFAs compounds distributed in different micro-polluted water systems, such as river water, lake water and seawater. Furthermore, applications in other fields can be considered as well, such as medical diagnostics where the sensor could be exploited for the monitoring of PFAs in body fluids.

## Figures and Tables

**Table 1:** Obtained values for the characterized sensors (with and without the MIP)-output signal normalized to 0 ppb (blank), by incubating solutions at increasing concentrations of PFOA in water solution (range 0–200 ppb).

PFOA Concentration [ppb]	POF-MIP Sensor	Bare POF Sensor (without MIP)
	$k_{norm}\pm \delta k_{norm}\;[a.u.]$	$k_{norm}\pm \delta k_{norm}\;[a.u.]$
0	1.0000 ± 0.0133	1.0000 ± 0.0138
0.1	0.9998 ± 0.0219	1.0004 ± 0.0111
0.2	0.9888 ± 0.0122	--
0.5	0.9736 ± 0.0185	0.9955 ± 0.0105
1	0.9458 ± 0.0278	0.9983 ± 0.0120
200	0.9323 ± 0.0160	0.9922 ± 0.0435

**Table 2:** Hill parameters (D-shaped POF platform with MIP).

Sensor	k_start_ [au]	k_end_ [au]	K_Hill_ [ppb]	n	Red. χ^2^	Adj.R^2^
**D-shaped POF- MIP**	0.9979 ± 0.0078	0.9319 ± 0.0041	0.6011 ± 0.1229	1.9137 ± 0.8441	0.06586	0.9766

**Table 3:** Chemical parameters (D-shaped POF-MIP sensor).

	PFOA Detection in Water Solution (*c* << *K_Hill_* and *n*≈1)	
	*Hill Parameters*	*Values*
D-shaped POF with MIP	Sensitivity at low *c* (|Δ*k_max_*/K_Hill_|) [a.u./ppb]	0.11
	LOD [ppb] (3×*δk_start_*/sensitivity at low *c*)	0.21

**Table 4:** Chemical parameters (SPR-POF sensor).

	PFOA Detection in Water Solution	
	*Hill Parameters*	*Values*
SPR-POF with MIP	Sensitivity at low *c* (∆λ_max_/K_Hill_) [nm/ppb]	22.1
	LOD [ppb] (3×standard deviation of blank/sensitivity at low *c*)	0.13
